# Benefits of expressive writing in reducing test anxiety: A randomized controlled trial in Chinese samples

**DOI:** 10.1371/journal.pone.0191779

**Published:** 2018-02-05

**Authors:** Lujun Shen, Lei Yang, Jing Zhang, Meng Zhang

**Affiliations:** School of Psychology, Xinxiang Medical University, Xinxiang City, Henan Province, P. R. China; Brown University, UNITED STATES

## Abstract

**Purpose:**

To explore the effect of expressive writing of positive emotions on test anxiety among senior-high-school students.

**Methods:**

The Test Anxiety Scale (TAS) was used to assess the anxiety level of 200 senior-high-school students. Seventy-five students with high anxiety were recruited and divided randomly into experimental and control groups. Each day for 30 days, the experimental group engaged in 20 minutes of expressive writing of positive emotions, while the control group was asked to merely write down their daily events. A second test was given after the month-long experiment to analyze whether there had been a reduction in anxiety among the sample. Quantitative data was obtained from TAS scores. The NVivo10.0 software program was used to examine the frequency of particular word categories used in participants’ writing manuscripts.

**Results:**

Senior-high-school students indicated moderate to high test anxiety. There was a significant difference in post-test results (*P* < 0.001), with the experimental group scoring obviously lower than the control group. The interaction effect of group and gender in the post-test results was non-significant (*P* > 0.05). Students’ writing manuscripts were mainly encoded on five code categories: cause, anxiety manifestation, positive emotion, insight and evaluation. There was a negative relation between positive emotion, insight codes and test anxiety. There were significant differences in the positive emotion, anxiety manifestation, and insight code categories between the first 10 days’ manuscripts and the last 10 days’ ones.

**Conclusions:**

Long-term expressive writing of positive emotions appears to help reduce test anxiety by using insight and positive emotion words for Chinese students. Efficient and effective intervention programs to ease test anxiety can be designed based on this study.

## Introduction

Test anxiety is a form of academic stress that is characterized by a feeling of nervousness before or during an exam [1]. Test anxiety is composed of two dimensions: worry and emotionality [2, 3]. Worry is the cognitive component of test anxiety reflecting debilitating thoughts and concerns, such as comparing self-performance to peers, considering the consequences of failure, low levels of confidence in performance, excessive worry over evaluation, causing sorrow for one’s parents, or feeling unprepared for tests [4–6]. Emotionality refers to heightened physiological symptoms stemming from arousal of the autonomic nervous system and associated affective responses, such as increased galvanic skin response and heart rate, dizziness, nausea, or feelings of panic [4, 5, 7]. Excessive test anxiety can affect learning, academic performance, and psychological health [8–10].

Test anxiety has been much researched in many countries, suggesting that this is a widespread problem in school, and it occurs among students of all ages [11–13]. Test anxiety is pervasive across geographic and cultural boundaries. Levels of test anxiety are reported to vary across cultures [14, 15]. Research has shown that 45.9% of senior-high-school students in China suffer from severe test anxiety [16].

Scholars have begun to attend more to the development of interventions aiming to reduce test anxiety. Researchers in many countries are seeking an ideal intervention program to help alleviate this problem. Ergene reported 22 existing interventions [17]. A meta-analysis by Lu et al. examined the effectiveness of certain intervention methods devised in China for test anxiety, such as cognitive therapy, psychoanalytic therapy, behavior therapy, and pharmacotherapy. The effect sizes of these methods were 0.70, 0.87, 2.38, and 1.99, respectively. These methods can reduce levels of test anxiety, however the most effective methods take the longest time or have higher requirements for the services of a psychological counselor [18]. There are limitations in the extent to which these methods can be generalized to more and more students. Given the drawbacks of these conventional interventions, scholars have begun seeking new approaches. Expressive writing may be one such approach—it has been found to significantly reduce levels of test anxiety and is rather easily implemented [19].

Expressive writing refers to the technique of writing a deep and meaningful piece about a personally significant topic, such as a traumatic or troubling event [20]. People’s reactions towards their own emotional responses, when experiencing stress, difficulty or failure, are important for their psychological adjustment. Indeed, greater self-reassurance is related to better psychological health [21]. Studies have shown not only that expressive writing can substantially improve a person’s emotional state via reduction of anxiety and depression [22], but also improve one’s social relationships and increase one’s feelings of well-being, happiness [23], and self-efficacy, as well as effectively promote one’s psychological health [22, 24–26]. Expressive writing has been shown to lead to improvements in both physical and psychological health in patients [27–29]. Joan attempted to translate the expressive writing intervention into a community-based intervention in the form of a videotaped program for rheumatoid arthritis patients. The results indicated that the videotape program instructing patients through the intervention at home was feasible, but achieving effectiveness in the community is very difficult. [30].

Expressive writing has been used to reduce levels of test anxiety or improve exam performance. Ramirez and Beilock asked American students in an experimental group to openly express their thoughts and feelings regarding math problems that they were about to perform. They noted a significant improvement in exam scores in the experimental group at a later assessment, and that the use of words related to anxiety, cause, and insight in their writing was positively related to math performance [31, 32]. Furthermore, Zhang et al. discovered, in their study on Chinese students, that writing to express negative emotions can increase participants’ test results in English courses [33]. However, most of the interventions were carried out just before exams or in a laboratory setting [31, 34, 35]. Some students suffer from chronic anxiety in their everyday lives, and may have a long history of suffering the deleterious effects of test anxiety. An intervention with only a small number of sessions might not be sufficient to have a long-term effect on the reduction of test anxiety. Knowles found that a short-term intervention has no effect in Asian culture [36]. Some researchers have revealed that a four-week intervention can effectively reduce the anxiety or improve the well-being of Chinese people [37]. Therefore, our study planned to carry out an intervention with 30 daily sessions, considering the school teaching plan, to help students to reduce their test anxiety.

With the rise of positive psychology over the past few decades, researchers have begun to pay more attention to the possible benefits of expressive writing on positive themes. Indeed, expressing positive emotions in writing can help to guide individuals’ attention to the positive aspects of a situation, aiding them in discovering the positive meaning of that event, and improving their emotional-regulation ability. This is not only a cost-effective method of treating anxiety and depression [17, 38–40], but also leads to improvements in mental flexibility and the ability to handle stress [41, 42]. People In western cultures, prone to use more insight and negative emotion words in expressive writing [31, 43]. At the same time, in eastern culture, people are more likely to suppress negative emotions than positive emotions [44]. Expressing negative emotions reveals one’s vulnerability, so people in eastern culture tend to prefer to express positive emotions, and prefer indirect and nonverbal modes of communication to explicit disclosure [45]. Hence, allowing Chinese students to write and express their positive emotions is more in accordance with the culture.

The National Higher Education Entrance Examination (*Gaokao*), a Chinese-specific examination, is considered the most important exam of a student’s life; indeed, the results are often considered to have a major impact on a student’s future. For that reason, Chinese students tends to experience rather high degrees of test anxiety when facing this examination [16, 46]. Therefore, it would be interesting to determine whether training in expressive writing of positive emotions would produce changes in test anxiety in senior-high-school students. We therefore hypothesized that expressive writing of positive emotion would reduce the test anxiety levels of senior-high-school students. This will provide some preliminary evidence for an effective intervention for test anxiety in this age group.

## Methods

### Participants and procedure

**Fig 1:**
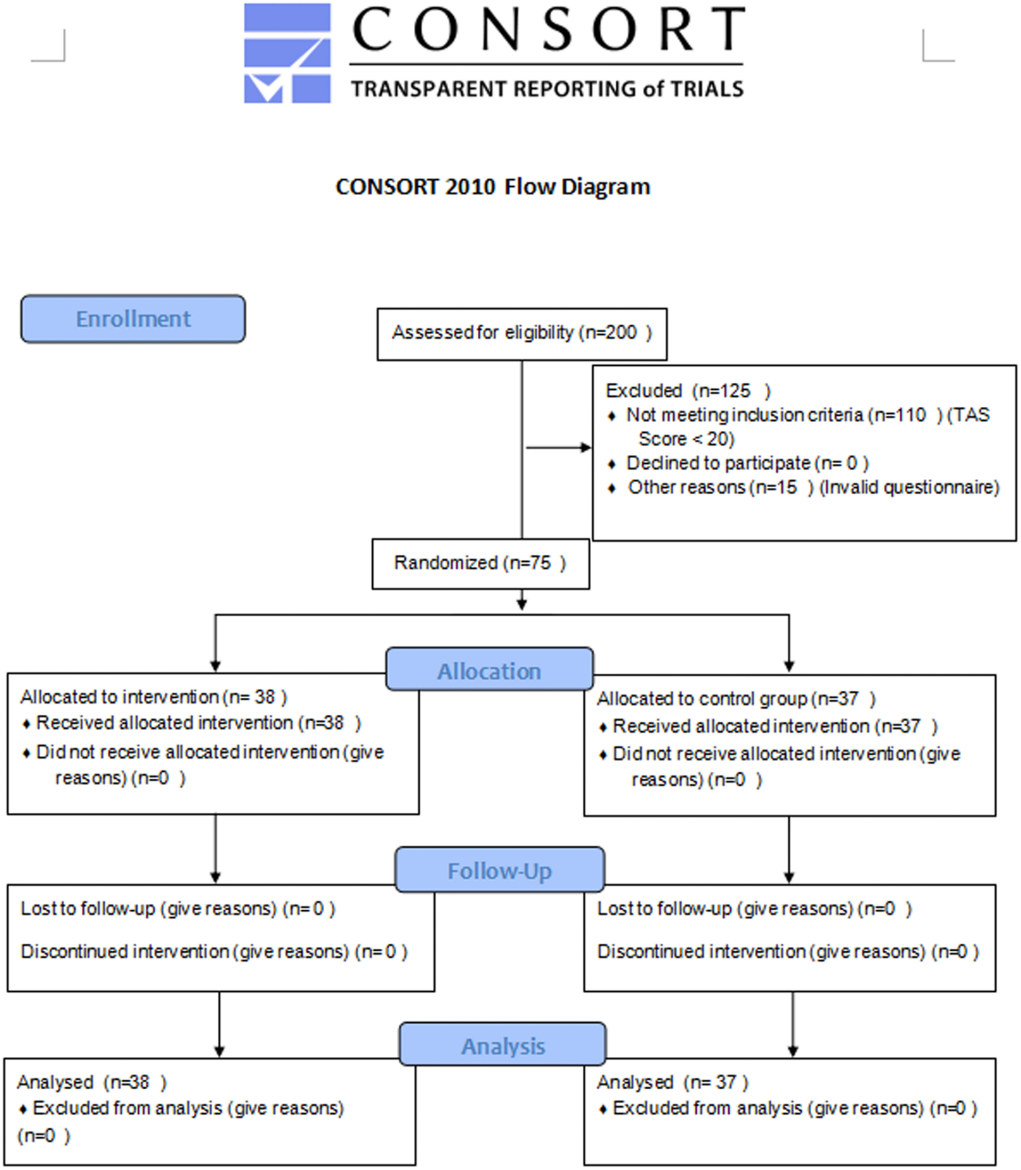
CONSORT flow diagram.

The participant recruitment and follow-up was from late April to early June. We randomly selected 200 students in second grade from three senior-high schools in Xinxiang city, and asked them to complete Sarason’s Test Anxiety Scale (TAS) in their classrooms. One hundred and eighty-five valid responses were collected (effective response rate 92.5%). A total of 75 students (21 males, 54 females, mean age of 16.84) with severe test anxiety were recruited. These students were divided into two groups by the high school teacher according to random numbers on a computer: the odd numbers were assigned to the expressive writing (EW) group, and the even numbers to the control writing (CW) group. The result revealed no statistically significant difference between the TAS results of the two groups prior to the experiment (*F* (1, 73) = 0.62, *P*> 0.05). The seventy-five participants’ demographic information (gender and age groups) is shown in Table 1. This research was approved by the Ethics Committee of Xinxiang Medical University. Researchers obtained written consent from students, and verbal consent from students’ parents. As it’s not convenient for researchers to meet the scattered-living parents, we informed them about the content by telephone, and recorded the time and each parent’s attitude. Only those parent-permitted students were included in our experiment. The whole process accompanied by students’ teachers. Please see the CONSORT flow diagram of the progress of participants throughout the trial in Fig 1. This research was registered in Chinese Clinical Trial Registry, and the registration number was ChiCTR-IOR-16007935 (URL: http://apps.who.int/trialsearch/Trial2.aspx?TrialID=ChiCTR-IOR-16007935).

**Table 1:** Baseline demographic characteristics of 75 participants.

Intervention	Gender		Age group		
	Male	Female	16 Y/O.	17 Y/O.	18 Y/O.
**EW Group**	11	27	8	27	3
**CW Group**	10	27	10	24	3

In the expressive writing (EW) group, participants were instructed to write about the positive emotions they felt every day, for 20 minutes at a time, consecutively for 30 days. We also asked participants to write their evaluations of the intervention at the last time. In the control writing (CW) group, participants were instructed to write, with the same frequency and duration as the EW group, about their daily activities. After the end of the writing, a second TAS was administered to both the EW group and the CW group. The data analyst was blinded to intervention status.

To reduce the loss of participants to follow-up and to ensure that they completed the activity each day, the researchers informed them of the requirements of this study before the experiment and provided each participant with pay. No students dropped out of the study. Moreover, with the consent of each participant, the researchers checked their completion of the task on a weekly basis.

### Measures

The data were collected from the TAS and students’ writing manuscripts. The TAS was developed by Sarason in 1978 [47]. In total, the TAS comprises 37 items, and each item is scored dichotomously (yes = 1, no = 0). The Cronbach’s α of the measurements was 0.84. Participants complete the questionnaire based on their actual situation for each item. A total score of 12 or less is considered to indicate low test anxiety, 12–20 is considered moderate anxiety, and 20 or higher indicates severe anxiety. A total score of 15 or higher indicates that the student has a sense of obvious discomfort.

Qualitative data were obtained from summaries of 38 students’ (EW group) writing manuscripts that were chosen from the first 10 days and the last 10 days. We examined the counts of particular code categories used in participants’ writing manuscripts with the Qualitative Solutions and Research International software program NVivo10.0.

## Quantitative results

### Descriptive statistical analyses

Participants’ TAS scores are described as follows. In general, students in this study indicated moderate to severe test anxiety (*M* = 18.05, *SD* = 6.08). Approximately 72.43% of students whose scores were 15 or higher had a sense of obvious discomfort (*M* = 20.84, *SD* = 4.38), and 42.86% of students whose scores were 20 or higher indicated experiencing severe test anxiety (*M* = 23.89, *SD* = 3.40).

### Comparison of TAS Scores by group and gender

As pre-test TAS scores may have an impact on post-test TAS scores, we therefore used analyses of covariance (ANCOVA) to accurately evaluate the impact of group (EW group, CW group) and gender (male, female) factors on post-test TAS scores. The dependent variable was post-test TAS scores, the fixed factors were group and gender, and the covariate was pre-test TAS scores.

**Fig 2:**
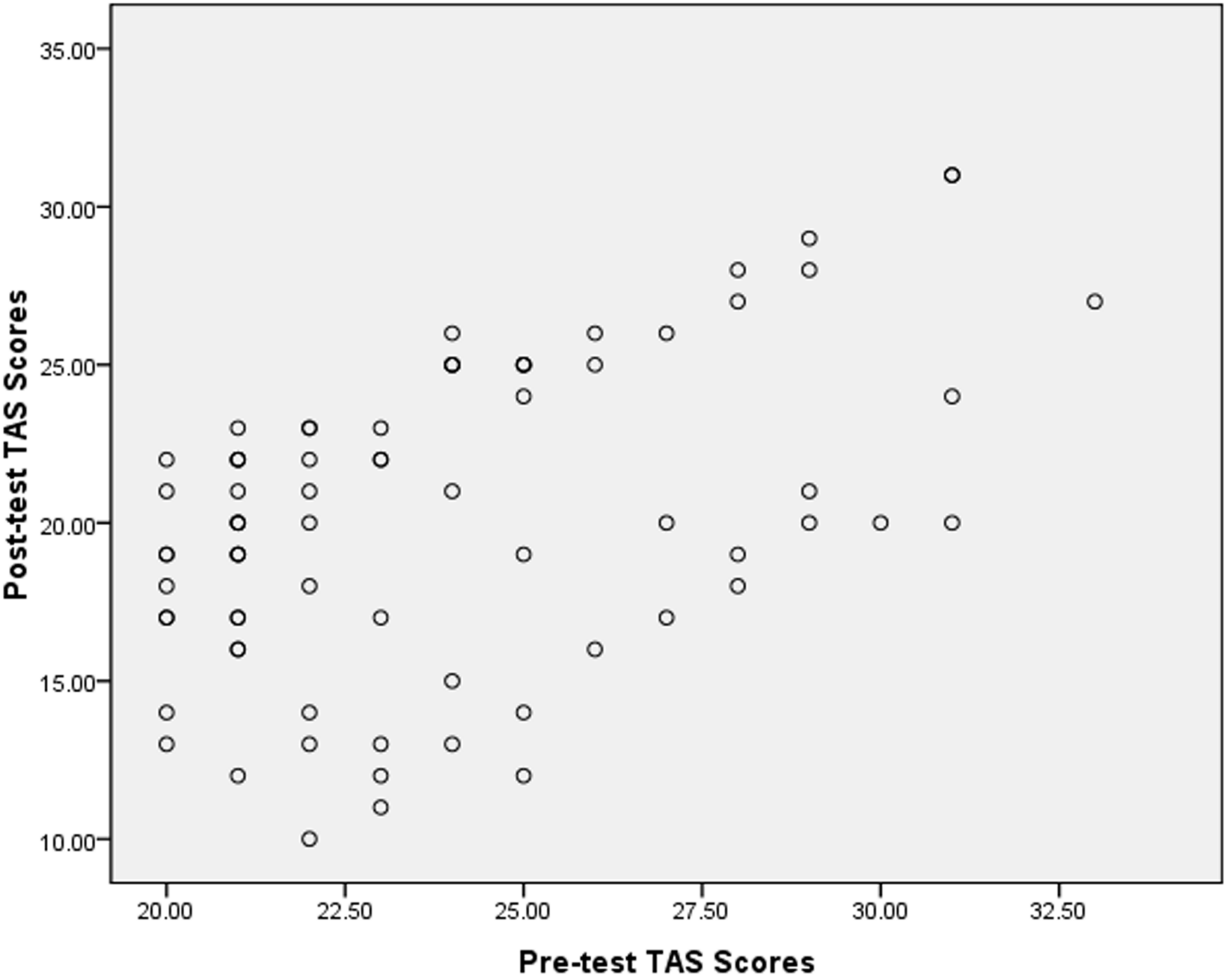
Scatterplots of the pre-test and post-test TAS scores.

To confirm whether the pre-test TAS scores were suitable for use as a covariate, the scatterplots of the pre-test and post-test TAS scores were examined (Fig 2)

Fig 2 shows that the pre-test and the post-test TAS scores had a linear relationship, and the slopes were basically the same. From this, we can initially confirm that the pre-test TAS scores can be used as a covariate (see Table 2).

**Table 2:** ANCOVA results for pre-test TAS scores.

Dependent Variable: post-test TAS scores					
Source	Type III Sum of Squares	df	Mean Square	F	Sig.
**Corrected Model**	1368.37^a^	4	342.09	62.33	0.00
**Intercept**	8.89	1	8.89	1.619	0.21
**Pre-test TAS Scores**	460.09	1	460.09	83.825	0.00
**Group**	745.40	1	745.40	135.80	0.00
**Gender**	12.03	1	12.03	2.19	0.14
**Group × Gender**	0.47	1	0.47	0.09	0.77
**Error**	384.21	70	5.489		
**Total**	32396.00	75			
**Corrected Total**	1752.59	74			

^a^ R Squared = 0.78 (Adjusted R Squared = 0.77)

Table 2 shows a significant main effect of group (*F* = 135.80, *P* < 0.001), with the average post-test TAS score of the EW group being significantly lower than that of the CW group; their difference value was 7.14 (*P* < 0.05). The effect size is 2.02, *P* < 0.01. No significant main effect of gender was found (*F* = 2.19, *P* > 0.05); the average post-test TAS score of males was non-significantly lower than that of females, with their difference value being 0.89. Furthermore, the interaction of group and gender in post-test TAS scores was non-significant (*F* = 0.09, *P* > 0.05).

To confirm whether the pre-test TAS scores were suitable for use as a covariate, another analysis was done involves generating the standardized residuals from a regression model where pre-test anxiety predicts post-test anxiety. And then use these standardized residuals as the dependent variable primary outcome in the ANOVA, the fixed factors were group and gender (Table 3)

**Table 3:** ANOVA results the standardized residuals.

Dependent Variable: the standardized residuals					
Source	Type III Sum of Squares	df	Mean Square	F	Sig.
**Corrected Model**	52.08^a^	3	17.36	58.87	0.00
**Intercept**	0.08	1	0.08	0.27	0.61
**Group**	40.34	1	40.34	136.81	0.00
**Gender**	0.68	1	0.68	2.29	0.14
**Group × Gender**	0.02	1	0.02	0.07	0.79
**Error**	20.94	71	0.30		
**Total**	73.01	75			
**Corrected Total**	73.01	74			

^a^ R Squared = 0.71 (Adjusted R Squared = 0.70)

Table 3 shows a significant main effect of group (*F* = 136.81, *P* < 0.001), with the average post-test TAS score of the EW group being significantly lower than that of the CW group. No significant main effect of gender was found (*F* = 2.29, *P* > 0.05); the average post-test TAS score of males was non-significantly lower than that of females. Furthermore, the interaction of group and gender in post-test TAS scores was non-significant (*F* = 0.07, *P* > 0.05). This result is consistent with the result of ANCOVA.

To further verify the effectiveness of the interventions, the Mann-Whitney U test, a non-parametric equivalent of the independent samples t test, was used. There was a significant difference between the difference values in TAS scores before and after the intervention of the EW and CW groups (*Z* = -7.38, Mann-Whitney *U* = 11.00, *P* <0.001). This indicates that participants who received the EW intervention experienced a significant decrease in their TAS scores compared to the control group. Furthermore, there was a non-significant difference between the difference values in TAS scores before and after the intervention of males and females (*Z* = -1.29, Mann-Whitney *U* = 458.00, *P* > 0.05). This indicates that male participants experienced a non-significant decrease in their TAS scores compared to female participants.

## Qualitative results

According to the researches made by Patton [48] and MacQueen [49], the codebook consisted of the followings the basic components: the codes, definition, guidelines for inclusion and exclusion criteria, and example text (see Table 4). A team of three researchers began by randomly selecting three manuscripts from the expressive writing data, and did not considering participants’ TAS scores. The copies of manuscripts were distributed to each researcher for independent analysis. Meetings were held at regular intervals to discuss discrepancies in coding until they reached agreement. Three manuscripts were randomly selected at a time. The team analyzed and coded the manuscripts until no new codes were identified. The remaining transcripts were coded by using the codebook as a guide. The team also consulted researches of Beilock and Knowles [32, 43]. Finally, a total of 5 main codes were identified (Table 5).

**Table 4:** Example of the cause code.

**Definition**: activities that motivate one’s positive emotions. **Inclusion criteria**: the activities which has enhanced students’ positive and enjoyable feelings, such as happiness and well-being. **Exclusion criteria**: activities that explicitly motivated one’s other feelings other than positive ones. **Examples**: Relaxation, Learning with others, Escaping from punishment, Receiving praise, Concentrating on learning.

**Table 5:** The codes that make up the codebook.

Code	Definition	Frequency ($\bar{x}$)	
		First 10 days	Last 10 days
**Cause**	Activities that motivate one’s positive emotions.	0.61	0.95
**Anxiety manifestation**	Statements about test anxiety.	5.81	2.79
**Positive emotion**	Statements about positive emotion.	1.81	6.25
**Insight**	Discovering your own thoughts and feelings.	2.15	4.31
**Evaluation**	Evaluation on the intervention		
Effectiveness	The intervention can reduce test anxiety		0.63
Writing frequency	The frequency of writing is acceptable.		0.84
Convenience	The intervention is easy to carry out.		0.76

*Note*. Frequency reflects the average counts of each code. The frequency of evaluation code only refers to the last manuscript.

### Cause

The participants said that their positive emotions can be most easily triggered by relaxation (such as eating delicious food or doing physical exercises) and learning with certain others. In addition, concentrating their mind on study, receiving praise from others, and escaping from punishment can also triggered positive emotion. At first, some students didn’t find anything to make themselves happy.

### Anxiety manifestation

This code contains two subcodes: cognitive component (Worry about learning and test) and emotional component (physiological and emotional responses about test anxiety). Although participants were required to write about positive emotions, they still showed the manifestation of anxiety. Emotionality included increased heart rate, dizziness, nausea, feeling of panic, and even falling into a swoon in their classroom. Worry mainly included feeling unprepared for tests, low levels of confidence in performance, exaggerating the impact of test results, especially causing guilt for their parents. During the early stages, twelve participants’ manuscripts were accompanied by suppression of anxiety, such as “men do not easily shed tears”.

### Positive emotion

This code contains two subcodes: cognitive component (Positive cognition of learning and test) and emotional component (Physiological and emotional responses to learning and test). The cognitive component of positive emotion included reducing learning stress, efficient learning, high levels of confidence in performance, gaining others’ approval, living up to parental expectations, and developing better. The emotional component included physical and mental pleasure, relaxation, and improving sleep quality.

“If I continue to work hard, I will be able to have an ideal score to return to my parents, and let them experience much glory and pride in front of their friends and relatives.”

Student A

### Insight

During later periods, participants increasingly used phrases such as “I feel,” “I think,” “I realized" to reveal their deep feelings and thoughts.

“When asked to write the positive emotion, I have to try my best to find happy events in my study. After several days, I begin to find that there is so much happiness in my life. I began to feel that my study is not only stressful and anxious, but also joyful and interesting.”

Student B

### Evaluations

Participants’ evaluations mainly focused on three sub nodes: effectiveness, frequency, and convenience.

#### Effectiveness

Twenty-four students said that they found the expressive writing of positive emotions was very helpful and that participating in the intervention was a correct choice. Eight students said that although they had experienced a reduction in anxiety as a result, they were still troubled by anxiety. Six students said that there were no changes in their levels of anxiety.

#### Writing frequency

Thirty-two participants said the writing frequency was acceptable, especially 8 of them combined with writing and language composition. On the other hand, six participants felt that writing every day was too frequent.

#### Convenience

Most students said that expressive writing only needs paper and pen, and can be carried out in the classroom or elsewhere. Twenty-nine students reported that with expressive writing one does not need to make an appointment with a psychological counselor, and the intervention requires only limited time and energy.

Among EW group, a higher use of positive emotion (*r* = -0.-0.54, *P* <0.01) and insight (*r* = -0.55, *P* <0.01) codes in the last 10 days manuscripts was associated with lower level of test anxiety. There was no significant relation between the anxiety manifestation, positive emotion expression, insight in the first 10 days and post-test TAS scores (*P* > 0.05). The results of Paired sample *t* test showed that there were significant differences in the positive emotion (*t* = -11.13, *P*<0.01), anxiety manifestation (*t* = 11.29, *P*<0.01), insight (*t* = -8.40, *P*<0.01_) codes between the first 10 days and the last 10 days.

## Discussion and conclusions

### The level of test anxiety in Chinese students

In general, students in this study indicated moderate to high test anxiety. This is consistent with previous findings from the literature [16]. Recently, the message “don’t let your children lose in the starting line” has rapidly become widespread in the educational and other fields in Chinese society. Chinese parents compete with others in early education and elementary education on behalf of their children [50, 51]. Chinese students have to acquire a great deal of knowledge before entering universities. Hence, Chinese students experience much greater learning pressure, and are prone to suffer from test anxiety and weariness [52]. For example, some participants may sometimes fell into a swoon in their classroom.

The writing manuscripts indicated that students’ test performance was related to their parents’ expectation and pride. This is consistent with the study by Xing et al. [53]. This may be related to culture. Chinese people exhibit collectivistic tendencies [54]. For example, the Chinese language does not have an expression comparable to “self-esteem” [55], and Chinese construal of the self is interdependent, meaning that the self is conceived as being embedded in the context of relationships with significant others [14, 56]. Chinese students are more likely to sacrifice personal goals, especially for their parents and family, and they reported significantly higher expectations, and greater concern about annoying their parents than American students. Chinese students’ interests and inner thoughts are restrained, and they tend to experience more conflict and anxiety. Pound found that Asian-American students reported significantly more test anxiety than their European-American peers [57]. Sansgiry and Sail found that American students indicated low to moderate test anxiety [58]. American culture exhibits individualistic tendencies [54]. It was reported that American students had a significantly stronger preoccupation about their capacity to face challenges in life than Chinese students [53].

### Expressive writing of positive emotions has a significant effect on test anxiety

The results of this study indicated that expressive writing of positive emotions could significantly reduce test anxiety levels among subjects. The qualitative research indicated that participants can reduce test anxiety and anxiety manifestation by increasing insight and positive emotion words. This is not consistent with the previous researches of people in Asian culture. Knowles found that Asian American cannot glean benefit from expressive writing [36]. When facing this pressure, Chinese people prefer to express their positive emotions and suppress their negative emotions [44]. For example, one participant wrote that “a man does not easily shed tears”. The writing about positive emotion was in accordance with Chinese culture. Participants increased their use of insight and positive emotion words across the long-term intervention, while there was no change of Asian American in short-term intervention [43]. Perhaps a long-term intervention is more suitable for the Chinese. Individuals spontaneously regulate their emotional experience when put in aversive situations [59–61]. In expressive writing, students with severe test anxiety pay increasing attention to their own deep feelings and thoughts by using insight and positive emotion words. During this period, they could have undergone a cognitive reappraisal of test anxiety through emotional expression. Cognitive reappraisal and emotional disclosure constitute a process of self–regulation [62]. When writing positive psychological content, the positive emotions induced by recalling pleasant experiences could help disperse students’ negative perceptions and emotions about their examinations, and direct their attention to the positive side of the situation. So the counts of anxiety manifestation decreased across the 30 days. This in turn could help them identify the positive meaning of the event, improving their ability to regulate their emotions, and eventually subtly reduce their anxiety towards tests.

Moreover, since students usually carried out their expressive writing in the evening, writing about their positive emotions may have helped them end the day on a happy note. This perhaps made it easier for them to feel relaxed and helped improve their sleep quality. A good night’s sleep would help them begin their day at school with more positive emotions and experiences. Thus, a virtuous circle might be formed, that over time would help reduce test anxiety and perhaps even improve learning efficiency [63].

The causes of positive emotion had Chinese characteristics, such as taking exercise and eating delicious food. In middle-school, physical education often gives way to testing, so students will feel very happy if they have a chance to exercise. Meanwhile, because of the limitations of time, students have little chance to eat their favorite foods. They would feel very happy when they eat delicious foods. Learning with certain others is also easy to motivate positive emotion, because the harmony and stability of their social networks has an important impact on people in Asian culture [43].

While the use of expressive writing for patients via home-based videotaped instructions may affect the degree to which patients adhere to interventions [30], our psychological counselor instructed students face to face at the beginning of writing. Students reported that expressive writing can be completed anywhere, do not require a psychological counselor each time, and places limited demands on time and energy. Expressive writing is a flexible, inexpensive, and convenient method.

### Expressive writing of positive emotions has a significant effect for both male and female students

Meanwhile, we can draw no conclusions on gender differences regarding the intervention on test anxiety. This means that expressive writing is effective for both females and males. Frattaroli’s meta-analysis also revealed no differences in the effect of gender on the expression of written emotion [64]. Females tend to be more emotionally sensitive and reactive, attentive, and affected by the surrounding environment, and good at expressing deeper feelings; thus, expressive writing might be expected to have a great effect on female subjects. Socialization practices encourage males to suppress their emotions including anxiety [15, 65]. Expressive writing of positive emotion can help males to express positive emotions and suppress negative emotions, and this is in accordance with Chinese traditions. Hence, anxiety in males is also reduced significantly.

## Conclusions

Overall, Chinese senior-high-school students indicated moderate to high test anxiety. Expressive writing can effectively reduce test anxiety by using insight words and expressing positive emotion. Since the timing of implementing this method is flexible and there are no specific requirements concerning location or the use of a psychological counselor, it can be considered an easy, inexpensive, and convenient method of managing test anxiety for students with severe test anxiety. Additionally, this intervention method can be used simultaneously with more than one student; given this high efficiency, it would be worth promoting in schools.

## Limitations

Although this study used a randomized design with a relatively large sample size, the limitations of this study should be considered. There are limitations in the extent to which the results can be generalized to other types of senior-high school students, as the results were obtained from a sample recruited only from Xinxiang City. Future research could focus on generalizing the results to a wider range of senior high school students. The study measured only the changes in test anxiety among students, and did not measure changes in their performance. This is the weakness of this research.

This limits our understanding of the students’ anxiety. The intervention over 30 days might be overly long and some students might be prone to fatigue. If there is no reward and informed consent, students might not persist for the entire intervention period. Future studies could consider reducing the writing frequency.

## Supporting information

S1 File CONSORT 2010 checklist. (DOC)

S2 File Informed consent for students. (DOC)

S3 File Record of informed consent for parents. (DOC)

S4 File Study protocol. (PDF)
