# Supplementary material for: Benefits of expressive writing in reducing test anxiety: A randomized controlled trial in Chinese samples
Source: PLoS One. 2018 Feb 5;13(2):e0191779. doi: 10.1371/journal.pone.0191779 (PMC5798770; doi:10.1371/journal.pone.0191779)
Supplement: S2 File — (DOC) [file pone.0191779.s002.doc]

**Informed Consent**

Dear friends:

Welcome you to participate in this experiment. In this experiment, you will be instructed to write special themes twice a week, for 20 minutes each time, consecutively for four weeks.

You can volunteer to participate in this experiment, and you can quit at any time without penalty. In order to protect your privacy, all information with Numbers instead of names to archive.

After this experiment, you'll get thirty yuan or equivalent items as a reward, dropping out is not compensated.

If you have read the conten, and agree to voluntarily participate in the study, please sign below.

Your name : Date:
