# Supplementary material for: Benefits of expressive writing in reducing test anxiety: A randomized controlled trial in Chinese samples
Source: PLoS One. 2018 Feb 5;13(2):e0191779. doi: 10.1371/journal.pone.0191779 (PMC5798770; doi:10.1371/journal.pone.0191779)
Supplement: S3 File — (DOC) [file pone.0191779.s003.doc]

**Record of Informed Consent for Parents**

| Students’ name | Parents’ name | Home address | Contact time | Parents’ attitudes | Teachers’ name |
| --- | --- | --- | --- | --- | --- |
|  |  |  |  |  |  |
|  |  |  |  |  |  |
|  |  |  |  |  |  |
|  |  |  |  |  |  |
|  |  |  |  |  |  |
|  |  |  |  |  |  |
|  |  |  |  |  |  |
|  |  |  |  |  |  |
