# Supplementary material for: Benefits of expressive writing in reducing test anxiety: A randomized controlled trial in Chinese samples
Source: PLoS One. 2018 Feb 5;13(2):e0191779. doi: 10.1371/journal.pone.0191779 (PMC5798770; doi:10.1371/journal.pone.0191779)
Supplement: S4 File — (PDF) [file pone.0191779.s004.pdf]

Protocol Synopsis

**Title :**	Benefits of Expressive Writing in Reducing Test Anxiety: a Randomized Controlled Trial in Chinese Samples.
**Purpose:**	To explore the intervention effect of expressive writing of positive emotions on test anxiety among senior-high-school students.
**Hypothesis :**	Expressive writing of positive emotions appears to help reduce test anxiety.
**Study Design:**	The study will employ a randomized controlled design in students with high test anxiety.
**Participants:**	From April 22 to April 28, 2015, we plan to randomly select 3 classes from three senior-high schools in Xinxiang city, and ask these students to complete the TAS in their classroom in the evening. Students with severe test anxiety will be recruited as participants. These participants will be divided into two groups by the high school teacher according to the random numbers on computer: the odd numbers were expressive writing (EW) group, and the even numbers were control writing (CW) group.
**Inclusion Criteria**	Participants must meet all of the following criteria.<br>①Students of grade 2 in senior high school.<br>②TAS score ≥20. The Test Anxiety Scale (TAS) was originally developed by Sarason in 1978, and then translated by Mr. Wang Caikang in 1999. A total score of 20 or higher indicates severe anxiety.<br>③Obtain informed consent from students and verbal informed consent from their parents.
**Exclusion Criteria**	Students may not be selected if any of the following criteria exist:<br>①Invalid questionnaire.<br>②TAS score ＜20.

	③Students or their parents refused to attend the trial.<br>④Students' expressive writing is not in conformity with the requirements.<br>⑤The trial seriously interferes with the participants' learning.
**Intervention:**	In the EW group, participants will be instructed to write about the positive emotions they feel every day, for 20 minutes at a time, consecutively for 30 days. In the CW group, participants will be instructed to write, with the same frequency and duration as the EW group, about their daily activities.
**Efficacy Measures:**	Change from pre-test scores to post-test scores.
**Participants Safety:**	If participants feel that their learning is affected by the trial, they can cease to participate in the trial.
**Statistical Analysis:**	The statistical analyst doesn't participate in the intervention.<br>The data analyst will use a 2 (intervention: EW or CW) × 2 (test: pre-test or post-test) mixed factorial ANOVA to analyze the difference in TAS scores, and use a 2 (Gender: Male or Female) × 2 (test: pre-test or post-test) mixed factorial ANOVA to analyze the difference in TAS scores.
**Ethics**	The trial must be according to the principles enunciated in Helsinki ethical principles declaration.<br>The trial has obtained informed consent from students and their parents, and has been approved by the Ethics Committee of Xinxiang Medical University on April 20.

书写表达缓解高中生考试焦虑的试验方案

试验负责单位：新乡医学院心理学系

试验负责人：申 鲁 军

审批部门：新乡医学院伦理委员会

审批时间：2015 年 4 月 20 日

1. 研究题目

书写表达对考试焦虑的随机对照干预研究

2. 研究背景

考试焦虑是指在一定的考试情境激发下，以担忧为基本特征，以防御或逃避为行为方式，通过不同程度的情绪反应表现出来的一种心理状态。中学生的考试焦虑发生率较高。为了有效地降低考试焦虑，国内外研究者进行了多种干预方法的探索。已有干预方法多属于行为疗法、认知疗法，传统干预方法效果并不理想。鉴于传统干预方法的弊端，研究者又开始寻找新的干预方法，研究表明书写表达是较为方便和实用的一种最新的考试焦虑干预方法。

书写表达是通过书写的方式表达与个人重要经历有关的感受，进而提升身心健康水平的一种心理干预方法。书写表达已经在临床上应用于各种身心疾病的康复和治疗。随着积极心理学的发展，研究人员发现积极主题的书写表达不仅能缓解个体的焦虑、抑郁等消极情绪体验，而且能提高心理弹性，增强压力应对能力等。然而目前国内运用书写表达干预考试焦虑的研究甚少。

鉴于以上，本研究采用书写表达积极情绪法对高中生的考试焦虑进行干预，以探讨其在缓解我国高中生考试焦虑方面的实际效果，为高中生过度考试焦虑的有效干预提供理论依据和实证参考。

3. 研究目的

考察书写表达对积极情绪对考试焦虑的干预效果。

4. 申报单位和研究单位

试验申办单位：新乡医学院心理学系

地址：河南省新乡市金穗大道 601 号

试验负责单位：新乡医学院心理学系

试验负责人：申鲁军　电话：3831813　E-mail: shenlj945@163.com

试验方案设计者：申鲁军

统计分析负责者：张晶

5. 试验设计

采用随机对照试验设计。

6. 被试选择

6.1 入选标准：

- 受试对象：高二学生。
- 采用由 Sarason 于 1978 年编制、王才康于 1999 年翻译的

Sarason 考试焦虑量表对学生进行施测，学生考试焦虑（TAS）得分：≥20。

- 男女不限。
- 学生签署知情同意书；获得家长的口头知情同意并记录在案。

6.2 排除标准：

- 无效问卷
- 考试焦虑（TAS）得分：＜20。
- 学生或家长拒绝参加试验。

6.3 淘汰标准

- 学生自己要求停止试验。为防止学生中途退出，给予被试一定的报酬。
- 书写表达不符合要求。
- 试验严重影响被试的正常学习。

7. 被试数及分组方法

7.1 被试数

本研究拟于 4 月 24 日从新乡市三所高中的高二年级各随机选取一个班的学生，于晚自习时在教室进行考试焦虑的测量，从中筛查出符合重度焦虑标准的学生作为试验被试。

7.2 随机分组方法

筛选出的被试，由高中老师借助电脑产生的随机数字表对被试进行编码，决定被试的分组，奇数进入实验组，偶数进入对照组。检测两组被试的 TAS 得分是否存在显著性差异。

8. 干预方法

8.1 实验组：书写表达积极情绪

让被试进行积极情绪的书写表达，指导语如下：在接下来的 30 天里，请你在每天晚上以日记的形式写下你今天体验到的快乐、幸福和乐观等积极情绪，例如：回忆并写下今天令你感到幸福的具体事情及当时的幸福心情。书写过程中请用心去思考和感受，每次书写时不需要考虑太多语法方面的问题，且书写过程中尽量不要中断，不要写成流水账。每次书写的时间为 20 分钟。

8.2 控制组：书写表达日常琐事

让被试进行日常琐事的书写表达，指导语如下：在接下来的 30 天里，请你在每天晚上以日记的形式写下你今天体验到的一些小事，不引发你的情绪感受的事件。书写过程中请用心去思考和感受，每次书写时不需要考虑太

多语法方面的问题，且书写过程中尽量不要中断，不要写成流水账。每次书写的时间为 20 分钟。

9. 疗效评估

观察指标：考试焦虑评价。

干预结束后，对被试再次进行考试焦虑（TAS）问卷施测。将干预前后的 TAS 进行对比，看是否下降。

10. 安全性评价

采用口头报告法，考察书写表达对被试学习状况的影响。如果被试报告学生状况受到影响，不愿继续参加试验，则停止参加试验。

11. 数据管理与统计分析

两次考试焦虑问卷测量回收后，由专门的数据处理人员进行数据处理。数据处理人员不参与试验过程。TAS 得分用均数和标准差描述，计数资料用例数描述。应用 SPSS 软件对进行两组被试测验前后 TAS 得分的差异性检验；对男女被试进行测验前后 TAS 得分的差异性检验。P 值小于或等于 0.05 被认为所检验的差别有统计学意义。

12. 试验总结报告

数据分析员分析完试验数据后，将结果提交给试验负责人，由试验负责人完成试验报告。

13. 伦理标准

试验方案必须在新乡医学院伦理委员会批准后方可实施。

试验前必须取得受试者的知情同意；因多数被试未年满 18 周岁，试验还需获取学生监护人的口头知情同意并记录在案。

研究符合《赫尔辛基宣言》

14. 资料的保存

试验负责人将资料保存完整，有固定地方存放并落锁保管，以备今后查看，资料保存至少 5 年。

15. 研究进度

2015 年 4 月 22-28 日：选取被试进行 Sarason 考试焦虑量表的前测，并设计书写表达积极情绪干预方案。

2015 年 5 月上旬---2015 年 6 月上旬：筛选高焦虑被试，随机分为试验组和控制组，进行干预对照研究。

2015 年 6 月上旬：对两组被试再次进行 Sarason 考试焦虑量表的测试。

2015 年 6 月中下旬：数据分析员进行数据分析工作。

2015 年 7 月至今进行论文撰写及投稿工作。
